# Myasthenia gravis complicated by pure red cell aplasia with clonal large granular lymphocytosis in the absence of thymoma: a rare case report and literature review

**DOI:** 10.3389/fimmu.2025.1367409

**Published:** 2025-02-03

**Authors:** Lijun Du, Yiping Liu, Qiaolin Zhou, Fang Xu

**Affiliations:** Department of Haematology, Mianyang Central Hospital, School of Medicine, University of Electronic Science and Technology of China, Mianyang, China

**Keywords:** pure red cell aplasia, myasthenia gravis, thymomas, large granular cells, autoimmune disorders

## Abstract

In 2013, a young woman during her early pregnancy was repeatedly hospitalized due to respiratory and swallowing difficulties. The pregnancy was terminated due to recurrent severe lung infections. She was later diagnosed with myasthenia gravis (MG) based on positive acetylcholine receptor antibodies. Her muscle weakness was subsequently well-controlled with pyridostigmine bromide, azathioprine, and prednisone. Notably, in the seventh year after her MG diagnosis (2021), the patient developed severe anemia (hemoglobin: 44 g/L). Bone marrow analysis revealed a rare combination of pure red cell aplasia (PRCA) with clonal expansion of large granular cells. Further examinations excluded thymoma. Considering the possibility of drug-induced PRCA, azathioprine was replaced with tacrolimus. Remarkably, the anemia resolved within 1 month, and her MG remained well-controlled. It is well-established that abnormal thymic hyperplasia within thymomas can alter the distribution and function of peripheral T lymphocytes, leading to the development of autoimmune diseases such as MG and PRCA. In this unique case without thymoma, we discussed the mechanisms and associations of PRCA with MG, medication, and clonal large granular T cells. This unique case highlights the unprecedented association of MG and PRCA without thymoma, underscoring the complexity of the disease spectrum. The patient’s subsequent successful delivery in June 2023 adds another dimension to the multifaceted clinical course, warranting attention and exploration into potential connections between these conditions.

## 1 Introduction

Myasthenia gravis (MG) is an autoimmune disorder characterized by muscle weakness and fatigue caused by impaired neuromuscular transmission. It primarily affects voluntary muscles, including those involved in breathing and swallowing. PRCA is a rare hematologic condition marked by a severe reduction or absence of erythroid precursors in the bone marrow, leading to anemia. Both MG and PRCA could be closely associated with thymoma, respectively. Reports indicated that approximately 10% of MG patients and 5%–13% of PRCA patients exhibited thymoma (1, 2). The intricate relationship between MG and PRCA has been well-documented, with both conditions frequently associated with thymoma (3–6). In the current literature, most reported cases of concurrent MG and PRCA are associated with thymoma. Besides our case, only three other cases without thymoma have been reported. Furthermore, a case with concurrent clonal large granular lymphocyte proliferation is unprecedented.

In this report, we present a case of concurrent MG and PRCA without an associated thymoma. We describe the clinical course, diagnostic workup, and treatment. We also discuss potential mechanisms between MG and PRCA, including the role of clonal large granular lymphocytes (LGL), immunosuppressive therapy, and autoimmune connections. Studying such rare cases is essential. Firstly, they present unique diagnostic and therapeutic challenges, highlighting the need for a multidisciplinary approach. Secondly, understanding the potential link between these autoimmune conditions could provide insights into their pathophysiology and improve patient management. Lastly, documenting and analyzing these cases can raise awareness and aid in the early recognition and treatment of similar presentations in clinical practice.

## 2 Case presentation

### 2.1 Diagnosis and treatment of myasthenia gravis

In April 2013, a young pregnant woman at 3 months’ gestation was admitted to a local hospital with complaints of respiratory distress, dysphagia, and coughing while drinking. Cerebrospinal fluid (CSF) analysis indicated cell–protein dissociation, raising suspicion of Fisher syndrome (FS). Despite receiving high-dose intravenous immunoglobulin treatment, her condition did not improve and relapsed.

In August 2013, she was admitted to our neurology department with worsening dysphagia. Neurological examination revealed the following: symmetrical nasolabial folds, normal strength for showing teeth and puffing cheeks, symmetrical forehead wrinkles, and strong eyelid closure without lagophthalmos. Muscle strength was grade 3 in both lower limbs and grade 5 in both upper limbs. The finger-to-nose and heel-to-shin tests were performed accurately bilaterally, but she could not perform tandem walking or the Romberg test. Ocular movements were full without lagophthalmos or gaze palsy, and no diplopia was elicited. She had strong and coordinated chewing, with no impairments in lip and teeth coordination. Thyroid hormone levels were within normal limits, and the autoantibodies were negative.

Owing to her critical condition and a history of repeated radiation exposure, prompt induction of labor was applied in September 2013 (34 weeks gestation). Over the next year, the patient experienced multiple severe respiratory infections, including tuberculous pleurisy, necessitating interventions such as tracheostomy, mechanical ventilation, antimicrobial and anti-tuberculosis treatments (isoniazid, rifampicin, pyrazinamide, and ethambutol), and bromhexine. Despite these measures, symptoms of muscle weakness, including respiratory distress, dysphagia, and speech weakness, persisted without improvement.

In August 2014, the patient underwent additional investigations at a higher-level hospital, revealing elevated acetylcholine receptor antibodies (AChR-Ab) (this information is from the medical records; the original data are not available) and electromyography (EMG) findings showing no abnormalities in motor conduction velocity (MCV) and F waves. Repetitive nerve stimulation (RNS) showed a decremental response, consistent with the diagnosis of oropharyngeal or generalized MG. Additionally, CSF protein levels and cell counts were normal on two separate occasions, thereby ruling out FS. Chest computed tomography (CT) revealed no thymoma. The patient had a negative neostigmine test result. Because of poor response to monotherapy with pyridostigmine bromide, she was treated with oral azathioprine 150 mg once daily and prednisone 15 mg in addition to pyridostigmine bromide, resulting in good control of symptoms, with noticeable alleviation of respiratory distress, dysphagia, and speech weakness. The patient could engage in light physical labor without recurrence of severe pulmonary infections.

### 2.2 Onset of pure red cell aplasia

Until 3 November 2021, the patient was readmitted to our hospital’s gastroenterology department with complaints of dizziness, fatigue, and dark stools, without respiratory or swallowing difficulties. Neurological examination revealed no ptosis or facial muscle weakness. However, there was evidence of limb muscle weakness and dysphagia, with symptoms exhibiting a diurnal variation, being milder in the morning and worsening by evening, and exacerbating with exertion. The patient exhibited no symptoms of acute abdomen, such as abdominal pain, acid reflux, heartburn, vomiting, or diarrhea. Abdominal examination revealed no tenderness, rebound tenderness, or abdominal muscle rigidity, and Murphy’s sign was negative. Gastrointestinal endoscopy revealed no evidence of bleeding or tumors. The fecal occult blood test was negative and both transaminase and bilirubin levels were within normal limits. Laboratory tests revealed red blood cells (RBCs) 1.4 × 10^12^/L, hemoglobin (HGB) 44 g/L, mean corpuscular volume (MCV) 103.6 fL, reticulocytes (RET) 0.0024 × 10^12^/L, and erythropoietin (EPO) level 3,739 mIU/L. Bone marrow smear indicated reasonable hematopoiesis with almost no erythroid series. Upon transfer to our department for further diagnosis and treatment, bone marrow biopsy revealed a hematopoietic-to-adipose tissue ratio of approximately 0.6:1, a granulocyte–erythrocyte ratio of approximately 10:1, reduced erythropoiesis, and overall diminished bone marrow hyperplasia primarily characterized by decreased erythropoiesis. Ferritin remained elevated at 3,119.16 ng/mL. Folate and vitamin B12 were normal, and the Coombs test was negative. Paroxysmal nocturnal hemoglobinuria (PNH) testing was negative. Viral markers (hepatitis B virus, hepatitis C virus, cytomegalovirus, human immunodeficiency virus, Epstein–Barr virus, and parvovirus B19) were negative. The common autoimmune disease-related antibodies, such as those associated with rheumatoid arthritis, systemic lupus erythematosus, Sjögren’s syndrome, systemic sclerosis, polymyositis, and primary biliary cirrhosis, were all negative. Flow cytometry of bone marrow and peripheral blood revealed a few large granular lymphocyte (LGL) cells: 9.2% in the bone marrow expressing cluster of differentiation (CD) 2, CD3, CD7, CD8, T-cell receptor (TCR) α/β, TCRγ/δ, and partially expressing CD57; 5% in peripheral blood expressing CD2, CD3, CD7, CD8, TCRα/β, and TCRγ/δ. Peripheral blood gene testing showed positive TCRγ gene rearrangement, with no mutations in STAT3 and STAT5b genes. Based on the comprehensive examination results, the diagnosis of pure red cell aplasia (PRCA) with concomitant clonal expansion of large granular T cells was established. Gastrointestinal bleeding and hemorrhagic anemia were ruled out. Repeat AChR-Ab showed a significant increase (a result of 3.91 nmol/L; normal range, <0.45 nmol/L), while the anti-muscle-specific kinase antibodies (MuSK-Ab), anti-connexin antibody (Titin-Ab), receptor lanny alkali-resistant calcium release antibodies (RyR-Ab), and anti-low density lipoprotein receptor-associated protein 4 antibody (LRP4-Ab) were normal. Repeat EMG showed negative RNS.

### 2.3 Outcome and follow up

Upon considering a diagnosis of PRCA, azathioprine was discontinued, and the patient underwent treatment with a regimen comprising RBC transfusion, tacrolimus, and prednisone.

**Figure 1:**
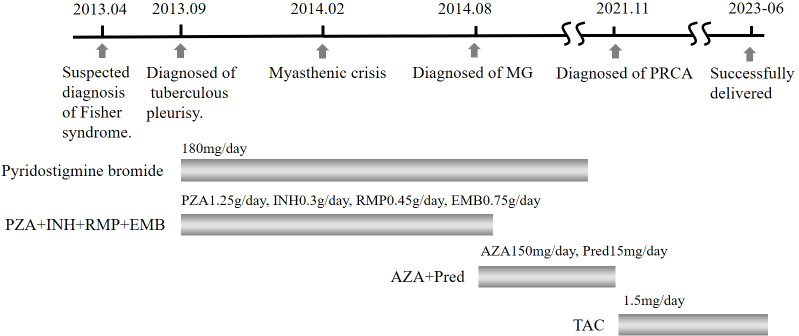
Timeline of primary diagnosis and pharmacological interventions. PZA, pyrazinamide; INH, isoniazid; RMP, rifampicin; EMB, ethambutol; AZA, azathioprine; Pred, prednisone; TAC, tacrolimus.

**Figure 2:**
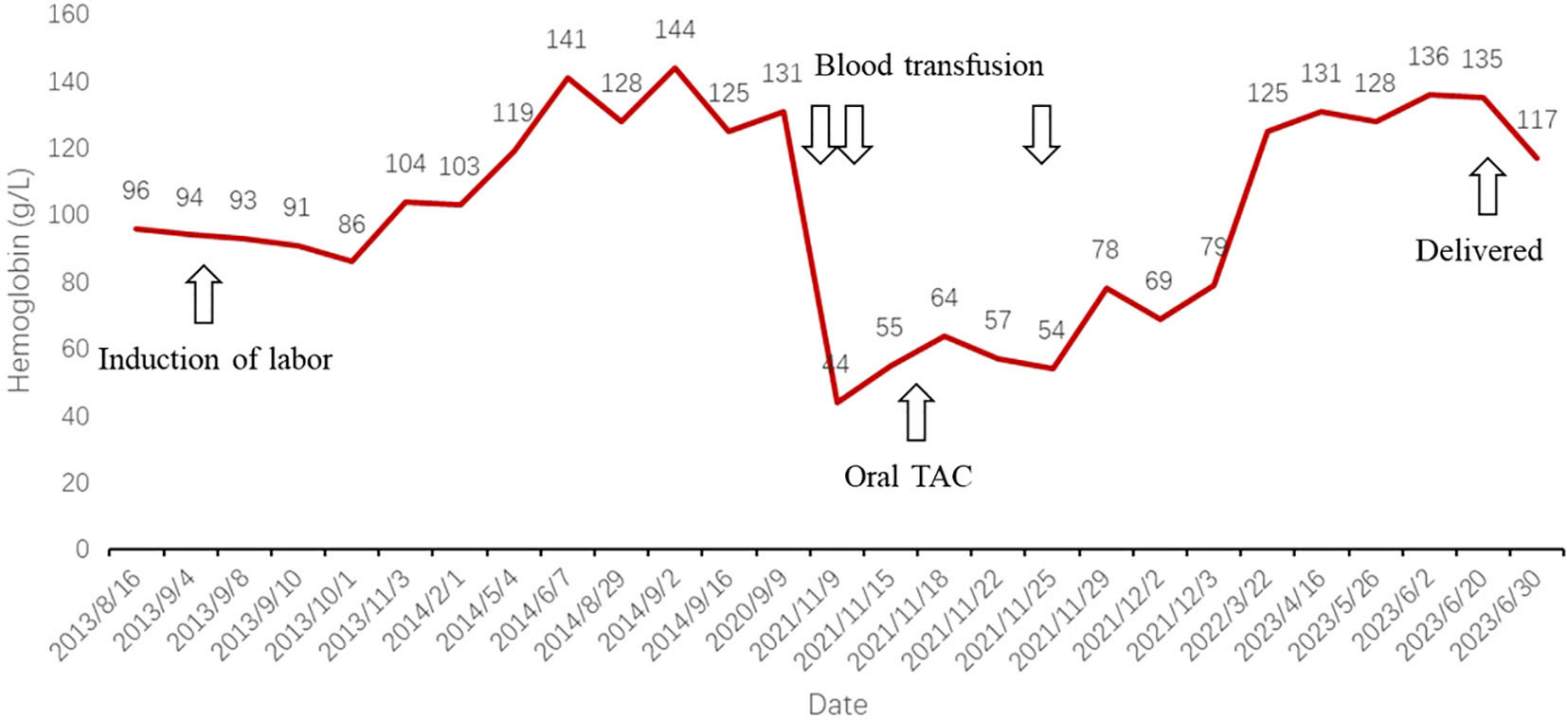
Line graph depicting trends in hemoglobin levels, significant events, and therapeutic interventions. TAC, tacrolimus.

Three months later, a blood analysis revealed normalized hemoglobin levels. She continued to adhere to a daily oral tacrolimus at 1.5 mg without visiting hematologists further. In October 2022, the patient became pregnant again. Throughout the pregnancy, she experienced no recurrence of MG or severe anemia. However, because of a COVID-19 infection, she had to undergo a cesarean section at 37 weeks and 4 days. The newborn was healthy, and both mother and child were discharged shortly thereafter. The patient’s summarized diagnostic and treatment course from 2013 to 2023 is depicted in **Figure 1**, while the trend of hemoglobin levels can be seen in the line graph in **Figure 2**.

## 3 Discussion

MG tends to worsen during pregnancy in approximately 30%–40% of female patients, especially during the first 3 months of pregnancy or postpartum (7). The patient in this case developed symptoms of MG at 3 months into pregnancy, and it is possible that pregnancy induced and exacerbated the MG symptoms. Because of the passage of time, it is unknown whether the patient had symptoms of MG, such as weakness or ptosis, before pregnancy. Approximately 8%–15% of MG patients have thymomas, and some may also have other associated conditions (8–13) such as PRCA, neuromyotonia, polymyositis, graft-versus-host disease (GVHD), and pseudo-obstruction, achalasia, among others. Secondary PRCA can have various causes (14–16), including autoimmune diseases, lymphoproliferative disorders, hematologic or solid tumors (such as thymomas), immune deficiencies, ABO-incompatible hematopoietic stem cell transplantation, viral or bacterial infections, and medications (e.g., recombinant human erythropoietin, immunosuppressive drugs, antibiotics, and antiviral drugs). A search of the PubMed database revealed over 100 reports of concurrent MG, PRCA, and thymoma. However, reports of MG with PRCA in the absence of a thymoma are extremely rare. Aside from the case reported in this paper, only three similar cases have been identified (17–19). Their medical history and treatment outcomes are summarized in **Table 1**.

**Table 1:** The characteristics of four cases with MG combined with PRCA without thymoma.

	Sex/age	Pre-existing condition	Sequence of onset	Parvovirus B19	AChR-Ab	Hemoglobin	Reticulocyte	Coombs test	PNH clone	Hematopoietic ingredients	Autoimmune antibodies	Thymoma	Bone marrow	Treatments	Outcomes
Aquilina et al.	M/65	Inexistence	PRCA preceded MG	IgM positive	Positive	6 g/dL	7×10^9^/L	Negative	Negative	Normal	Negative	Thymic cyst (based on PET-CT and histopathology)	Hypercellular marrow, reduction of erythroid precursor, myeloid hyperplasia	Packed cell blood transfusions, pyridostigmine, intravenous Ig	Remission
Nakamura et al.	F/47	SLE, isoniazid	MG preceded PRCA	IgM, IgG, and DNA all negative	Positive	5.8 g/dL	1×10^9^/L	NA	NA	Normal	Negative	Inexistence	Reduction of erythroid precursor, normal myeloid cells, and megakaryocytes	Pyridostigmine bromide, mizoribine, red blood cell transfusion, cyclosporine A, tacrolimus,	Remission
Mohri et al.	F/37	Hodgkin’s lymphoma, chemotherapy	Simultaneously	NA	Positive	NA	NA	NA	NA	NA	NA	Inexistence	Reduction of erythroid precursor	steroid, and pyridostigmine	Died of pneumonia
Our case	F/17	Pregnancy	MG preceded PRCA	DNA negative	Positive	4.4 g/dL	2.4×10^9^/L	Negative	Negative	Elevated ferritin levels	Negative	Inexistence	Overall diminished bone marrow hyperplasia primarily characterized by decreased erythropoiesis	Pyridostigmine bromide, azathioprine, red blood cell transfusion, tacrolimus, and prednisone	Remission, successful conception, and childbirth

PNH, paroxysmal nocturnal hemoglobinuria; PET-CT, positron emission tomography–computed tomography; SLE, systemic lupus erythematosus; NA, not available.

The rare case we are reporting occurred during a 7-year course of azathioprine treatment for the diagnosis of MG, and PRCA developed as a complication. Phenotypically atypical large granular lymphocytes with T-cell receptor (TCR) gene rearrangement were observed in bone marrow and peripheral blood flow cytometry. Several potential mechanisms for the development of PRCA in this case are proposed. First, we hypothesize that the onset of PRCA may be associated with the clonal proliferation of large granular lymphocytes. In a cohort of 47 PRCA patients at Mayo Clinic, LGL leukemia was the most common associated condition, with approximately 19% of patients being diagnosed with LGL leukemia (20). Even though this patient did not meet the criteria for a diagnosis of T-cell large granular lymphocytic leukemia (T-LGLL), clonal T-LGL cells were indeed present. The potential mechanism involves cytotoxic T-LGL cells whose surface TCR recognizes antigens presented by class I human leukocyte antigen (HLA-I) on red cell precursors, leading to the activation of their cytotoxic signals. Under normal circumstances, this cytotoxic signal is counteracted by inhibitory signals produced when HLA-I molecules on the surface of red cell precursors interact with their inhibitory receptors. However, during the differentiation process of red cell precursors, the expression of HLA-I class molecules is physiologically downregulated (21). The patient was not evaluated with bone marrow and peripheral blood flow cytometry when initially diagnosed with MG; thus, it is unknown when these clonal T-LGL cells first appeared.

Second, the occurrence of PRCA may be drug-induced. The patient had been on azathioprine treatment for MG for an extended period before the development of PRCA. There have been multiple cases reported where PRCA occurred after kidney transplantation or in patients with Crohn’s disease treated with azathioprine (22–26). It is believed that this might be related to the azathioprine metabolite 6-thioguanine (6TGN), which inhibits DNA synthesis and exerts direct cytotoxicity on erythroid precursor cells, especially in patients with reduced activity of thiopurine methyltransferase (27). The timing of bone marrow toxicity varies widely, from a few days to 11 years, with a higher incidence in the first 8 weeks (23). Therefore, the role of azathioprine as a contributing factor cannot be ruled out in this case. In addition, it has been reported that anti-tuberculosis drugs such as isoniazid and rifampicin may also lead to the occurrence of PRCA (17, 28–30). In such cases, hemoglobin levels typically recovered gradually after discontinuation of the drugs (30). Mizobuchi et al. speculated that T cell-mediated immune responses may be associated with the secondary development of PRCA (6). Proposed mechanisms include direct cytotoxic effects or metabolic interference impacting erythropoiesis, as well as immune-mediated inhibition of bone marrow erythroid progenitor cells (31, 32). The patient in this case underwent anti-tuberculosis treatment from September 2013 to August 2014, which included isoniazid and rifampicin. However, severe anemia occurred in November 2021, suggesting that it may not be related to these agents.

Most published cases of MG complicated by PRCA are associated with thymoma, and the pathophysiological mechanisms underlying the association of thymoma with autoimmune diseases or paraneoplastic syndromes remain unclear. Some researchers have reviewed the pathological and clinical features of 42 thymoma patients with paraneoplastic syndromes and speculated that the coexistence of thymoma with PRCA, MG, and other autoimmune diseases may be related to the generation, migration, and subsequent interaction of autoantigen-specific T cells with B lymphocytes outside the thymus (33). As for patients without thymoma, there may be an immunological connection between MG and PRCA. Reports indicate that patients with both MG and PRCA typically develop PRCA several years after the onset of MG, with only 1 out of 11 cases in a series where PRCA occurred before the onset of MG (34). This implies that MG may initiate a mechanism resulting in the onset of PRCA. Within the thymus, immature T cells undergo differentiation, clonal selection, and proliferation before being released into the circulation. When associated with thymomas, aberrant thymopoiesis within thymomas can modify the peripheral T-cell repertoire, potentially leading to the generation of autoantigens and the development of thymoma-associated autoimmune diseases (35). However, this patient did not present with a thymoma, necessitating further clinical observation and mechanistic research in similar cases to elucidate the connection between MG and PRCA.

After more than 8 years, the patient became pregnant again and successfully delivered, despite long-term treatment with tacrolimus and prednisone. This pregnancy did not exacerbate MG symptoms, aligning with current clinical experience (1). Authoritative consensus recommends treatment of MG during pregnancy primarily with pyridostigmine and glucocorticoids, with minimal impact from immunosuppressive therapy on fertility (1). In this case, combination immunosuppressive therapy was necessary due to concurrent PRCA. Tacrolimus is widely used to prevent rejection following solid organ transplantation. Although pregnancies in organ transplant recipients are considered high risk, more than 14,000 such pregnancies have been reported (36). While pregnancy can affect the whole blood concentration of tacrolimus, it does not impact its free concentration (36); thus, dose adjustments are not necessary during pregnancy. Our patient experienced a smooth pregnancy and delivered a healthy newborn, suggesting minimal fetal side effects from tacrolimus. This case provides valuable clinical insights, although further observations are warranted.

MG could potentially trigger other autoimmune disorders, such as PRCA in the case described here. Immunosuppressive agents may also contribute to the development of PRCA, necessitating close monitoring of blood parameters during treatment. In this rare case, the coexistence of MG and PRCA, along with an increase in clonal LGLs, warrants continued surveillance to determine whether the disease undergoes further evolution under well-controlled MG and PRCA conditions.

## Data Availability

The original contributions presented in the study are included in the article/supplementary material. Further inquiries can be directed to the corresponding author.
